# Stochastic simulation of soil moisture dynamics in farmland in the eastern region of the Songnen Plain

**DOI:** 10.1371/journal.pone.0318161

**Published:** 2025-01-30

**Authors:** Bo Meng, Jin Wang, Fanxiang Meng, Ennan Zheng, Tianxiao Li, Bowen Tang

**Affiliations:** 1 School of Hydraulic and Electric Power, Heilongjiang University, Harbin, Heilongjiang, China; 2 School of Water Conservancy and Civil Engineering, Northeast Agricultural University, Harbin, Heilongjiang, China; Jiangsu University, CHINA

## Abstract

Soil moisture is the core of the hydrological cycle in agroecosystems, and most of the studies on soil moisture dynamics modeling adopt deterministic research methods, which are not well suited to study the hydrological processes in agricultural fields under changing conditions. Therefore, the present study adopts a stochastic approach to reveal the distribution characteristics of soil moisture in agroecosystems under the effects of soil, climate, vegetation, and other influencing factors. Using soil moisture and precipitation data and based on a stochastic model of soil moisture dynamics, the point-scale soil moisture dynamic characteristics and soil moisture probability density function of farmland systems in the Songnen Plain region were investigated. The soil moisture of maize in the study area showed a certain degree of stochasticity, and the curve characteristics of the probability density function of soil moisture *p(s)* obtained from the simulation were very close to those of the measured *p(s)*. It shows that the stochastic model can effectively simulate the probability density function of soil moisture in the study area, which can provide a theoretical basis and scientific method for efficiently using soil and water resources in the area.

## 1. Introduction

Soil water is generally defined as the moisture of unsaturated soil from below the surface to the air-packed zone above the groundwater surface, and is the key link between surface water and groundwater [1]. As an important water resource, soil water is one of the important components of the soil and ecohydrological cycle, plays an important role in the formation, transformation, and consumption of water resources, and has a large impact on soil erosion, material transport in the soil-vegetation-atmosphere transporter (SPAC), and soil formation processes [2–4] In farmland ecosystems, soil water is the carrier of nutrient cycling and is the main source of crop water, its distribution condition directly affects crop development and yield and is one of the main driving forces for crop growth [5–7]. However, the distribution status of soil water is affected by a variety of factors such as rainfall, vegetation types, geological conditions, and evaporation, resulting in very complex soil moisture dynamics, and soil moisture dynamics show a large degree of randomness. Therefore, to describe the soil water dynamics under the nonlinear effects of soil, vegetation, and climate factors, it is necessary to adopt a stochastic approach to the stochastic simulation of the soil water dynamics model.

Most of the early soil moisture models were numerical descriptions of empirical or semi-empirical nature, and the research on soil moisture dynamics was only focused on linear or nonlinear relationships [8], which mainly utilized the time series analysis method to extract and decompose the periodicity, trend, and stochasticity of the law of soil moisture dynamics, and to establish simple stochastic models [9]. Eagleson [10] proposed the stochastic concept to simulate the movement of moisture in the soil-vegetation-atmosphere continuum (SPAC) system in the 1970s and established a stochastic dynamics water balance model, but Eagleson’s theory is too complex, so it is not widely applied in the analysis of practical problems. Milly [11] assumed that the evapotranspiration of vegetation was independent of soil moisture, and established a stochastic model of soil moisture dynamics, in which a set of very parsimonious equations was used to describe the soil moisture balance on a small time scale. Rodriguez-Iturbe et al. [12] derived an analytical solution of the differential form of the soil water balance equation, i.e., the soil moisture probability density function, and analytically obtained the probability distribution of soil moisture at a point in space; evapotranspiration was considered to be a function of soil moisture and deep percolation was nonlinearly correlated with soil moisture in the Rodriguze-Iturbe model. Porporato et al. [13] proposed a soil moisture stress index to quantify the response of vegetation to water stress duration and water deficit, and applied it in the study of vegetation water stress under different climatic conditions. Laio et al. [14] improved the evapotranspiration term on the basis of the Rodriguze-Iturbe by introducing the hygroscopicity coefficient and wilting coefficient as two soil moisture parameters, which realized the accurate simulation of soil moisture movement of vegetation under water deficit conditions. Liu et al.[15] used the continuous monitoring data of soil moisture during the growing season in grassland in Qilian Mountain area as well as daily precipitation data, combined Laio’s improved stochastic model of soil moisture dynamics with Monte-Carlo simulation method to study the characteristics of the point-scale growing season soil moisture dynamics and soil moisture probability density function in grassland ecosystems of the shallow mountainous areas of the Qilian Mountains. Ridolfi et al. [16] considered the dependence of soil moisture on topographic conditions, and further developed the Rodriguze-Iturbe and Laio models, which investigated the relationship between the subsurface unsaturated boundaries and the climatic conditions, soil properties, and slope characteristics. Pan et al. [17] modified the Rodriguze-Iturbe model and used the modified Rodriguze-Iturbe model to analyze the soil moisture dynamics of irrigated farmland for winter wheat and maize in the North China Plain region, and found that there was no significant bimodal nature of the average soil moisture probability density function under the influence of precipitation fluctuations. Wang et al. [18] used a stochastic soil moisture balance model to simulate soil moisture as well as water balance in the Loess Plateau region, and the model was validated by monitoring the soil moisture data of acacia plantation forest in the Sheep Gap watershed of the Loess Plateau, in addition to exploring the impacts of rainfall pattern variations on soil moisture and water balance. Wu et al. [19] designed a comprehensive model integrating energy balance and water cycle processes, simulated using a randomly generated 100-year climate time series, analyzed the soil moisture dynamics underneath photovoltaic (PV) panels in an arid region as well as the changes in average soil temperature, and obtained the probability density distributions of soil moisture as well as temperature.

Although stochastic modeling of soil moisture dynamics has been applied to many different regions, little research has been reported for the black soil region of the Songnen Plain, and the accuracy of soil moisture collection in previous studies has been poor. Therefore, in this paper, we improved the accuracy of soil moisture data (collected day by day), and the main research objectives: (1) studying the characteristics and probability distribution of soil moisture at different depths in the black soil zone of the Songnen Plain; (2) Applying the Laio stochastic model to the black soil zone of the Songnen Plain and determining the sensitivity of the stochastic model to different parameters. The study’s results can provide a reference for crop cultivation in the black soil area of the Songnen Plain and provide theoretical guidance for the efficient utilization of soil water resources in farmland in the region.

## 2. Materials and methods

### 2.1 Situation in the study area

The study area is located in the southeast of the Songnen Plain at Harbin Wanjia Experimental Station (geographic location 126°37′E, 45°58′N, mean elevation 171.7 m), which has a temperate monsoon climate with four distinct seasons. The average annual temperature is 3.8–5.3°C, the average evaporation is 659mm, and the average precipitation is 534mm. The soil in the study area is dominated by black soil with high soil organic matter content, which is suitable for crop growth. The cropping system is annual, and the main crops are corn, soybean, and rice, of which corn is sown in early May and harvested in late September to early October.

### 2.2. Stochastic modeling of soil moisture dynamics

**Fig 1:**
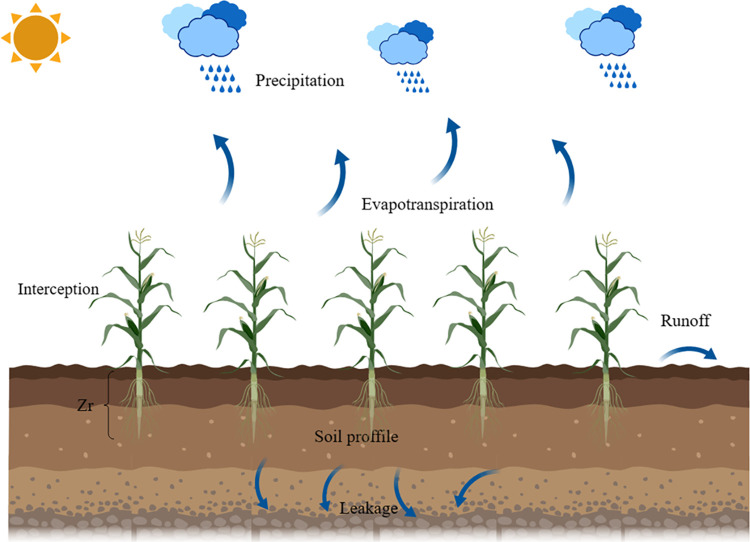
Schematic diagram of soil water balance.

The Laio model is based on the principle of conservation of soil moisture mass in the vegetative root zone and describes the daily-scale vertical mean water balance in the vegetative root zone during the growing season [20], The soil water balance profile is shown in **Fig 1**. Under simplified conditions that do not take into account lateral and vertical effects [21], the soil water balance in space on a daytime scale at a point is represented by a nonlinear stochastic ordinary differential Eq (1):

$$nZr\frac{ds}{dt}=\varphi [s(t);t]-\chi [s(t)]$$ (1)

φ[s(t);t]=R(t)−I(t)−Q[s(t);t] (2)

χ[s(t);t]=E[s(t)]+L[s(t)] (3)

Where *n* is the soil porosity; *Zr* is the rhizosphere depth; *s*(*t*) is the relative soil water content, *s*(*t*) = *θ*(*t*)/*n*, *θ*(*t*) is the volumetric soil water content; *φ*[*s*(*t*);*t*] indicates the input term to the water balance, which is the infiltration rate of rainfall (cm/d); *R(t)* is the rate of precipitation (cm/d); *I(t)* is the retention capacity of the vegetation canopy (cm); *Q*[*s*(*t*);*t*] is the rate of surface runoff (cm/d); *χ*[*s*(*t*);*t*] indicates the loss term of the water balance, which is the rate of loss of soil water (cm/d); *E*[*s*(*t*)] is the rate of evapotranspiration from the vegetation (cm/d); and *L*[*s*(*t*)] is the rate of deep percolation (cm/d).

Assumptions in the model include: the occurrence of a precipitation event is a series of point events in continuous time, obeying a Poisson process with frequency; a single precipitation event occurs instantaneously, and the depth of precipitation h produced by each precipitation event obeys an exponential distribution with a mean value of *α*(mm); the vegetation interception is a threshold value of *Δ* such that, if the depth of a precipitation event does not exceed this value, all of the precipitation is intercepted, and, if it exceeds this value, then the excess is the amount of water that actually reaches the soil; the soil is a uniformly distributed water storage medium with porosity *n* and depth *Z*_*r*_: when the soil has sufficient capacity to hold the water from a rainfall event, all of the infiltrated precipitation is converted to soil water; when the depth of the rainfall event exceeds the capacity of the soil, the excess is converted to surface runoff; the depth of infiltration is determined by the minimum depth of the precipitation event and by the water content of the soil at the time of occurrence of the precipitation event. When the soil is completely saturated (*s* = 1), runoff is generated [22], and the runoff process is considered to be dominated by dunne runoff [23]. Groundwater-soil water interactions and boundary flow infiltration are not considered in the model [24].

Soil evaporation and vegetation transpiration are not considered separately, The evapotranspiration loss term in the model is the sum of the two. When soil moisture rises from the moisture absorption coefficient to the wilting coefficient, there is only soil evaporation, and the rate of evapotranspiration increases slowly and linearly from 0 to *E*_*w*_(soil evaporation corresponding to the wilting coefficient); soil moisture rises from the wilting coefficient *S*_*w*_ to the start of the moisture stress point S^*^, the rate of evapotranspiration increases rapidly and linearly from *E*_*w*_ to *E*_*max*_ (the maximum amount of evapotranspiration); and the soil moisture starts from the start of the moisture stress point, and always maintains the maximum amount of evapotranspiration *E*_*max*_. The modeled evapotranspiration loss term *E(s)* as a function of soil moisture *s(t)* was as follows:

$$E(s)=\{\begin{array}{ll} 0 & 0<s\leq s_{h}\\ E_{w}\frac{s-s_{w}}{s_{w}-s_{h}} & s_{h}<s\leq s_{w}\\ E_{w}+(E_{\max}-E_{w})\frac{s-s_{w}}{s_{w}-s_{h}} & s_{w}<s\leq s^{*}\\ E_{\max} & s^{*}<s\leq 1 \end{array}$$ (4)

When the soil moisture is greater than the water-holding capacity of the field, seepage begins to occur and the seepage loss term *L*[*s*(*t*);*t*] can be expressed by the following equation:

$$L[s(t)]=\frac{K_{s}}{e^{\beta (1-s_{fc})}-1}[e^{\beta (1-s_{fcc})}-1]$$ (5)

Where *K*_*s*_ is the soil-saturated hydraulic conductivity; *β* is the pore size distribution parameter, *β* = 2b+4, and *b* is the soil pore aperture distribution index, which can be obtained from the soil moisture characteristic curve.

The effect of stochastic precipitation makes the solution of Eq (1) meaningful only in probabilistic form, and the solution of the probability density function of soil moisture can be derived from the Chapman-Kolmogorov forward equation by taking the *t* tends to infinity limit for the Chapman-Kolmogorov forward equation to obtain the following ordinary differential equation:

$$\frac{d}{ds}[\rho (s)p(s)]+\gamma \rho (s)p(s)-\lambda^{\prime }p(s)=0$$ (6)

Where *ρ(s)* is the soil moisture loss function and the general form of the soil moisture probability density function *p(s)* for the solution of Eq (6) is:

$$p(s)=\frac{c}{\rho (s)}e^{-\gamma s+\lambda^{\prime }\int \frac{du}{\rho (u)}},\;s\geq s_{h}$$ (7)

A specific expression for *p(s)* can be obtained from the soil moisture loss function *ρ(s)*:

$$p(s)=\{\begin{array}{ll} \frac{c}{\eta_{w}}{\left(\frac{s-s_{h}}{s_{w}-s_{h}}\right)}^{\frac{\lambda^{\prime }(s_{w}-s_{h})}{\eta_{w}}-1}e^{-\gamma s} & s_{h}<s\leq s_{w}\\ \frac{c}{\eta_{w}}{\left[1+(\frac{\eta }{\eta_{w}}-1)(\frac{s-s_{w}}{s^{*}-s_{w}})\right]}^{\frac{\lambda^{\prime }(s^{*}-s_{w})}{\eta -\eta_{w}}-1}e^{-\gamma s} & s_{w}<s\leq s^{*}\\ \frac{c}{\eta }e^{-\gamma s+\frac{\lambda^{\prime }}{\eta }(s-s^{*})}{\left(\frac{\eta }{\eta_{w}}\right)}^{\lambda^{\prime }\frac{s^{*}-s_{w}}{\eta -\eta_{w}}} & s^{*}<s\leq s_{fc}\\ \frac{c}{\eta }e^{-(\beta +\gamma )s+\beta s_{fc}{\left(\frac{\eta e^{\beta s}}{(\eta -m)e^{\beta s_{fc}}+me^{\beta s}}\right)}^{\frac{\lambda^{\prime }}{\beta (\eta -m)}+1}}{\left(\frac{\eta }{\eta_{w}}\right)}^{\lambda^{\prime }\frac{s^{*}-s_{w}}{\eta -\eta_{w}}}e^{\frac{\lambda^{\prime }}{\eta }(s_{fc}-s^{*})} & s_{fc}<s\leq 1 \end{array}$$ (8)

Where,

$$\eta_{w}=\frac{E_{\max}}{nZ_{r}}$$ (9)

$$\eta =\frac{E_{w}}{nZ_{r}}$$ (10)

$$m=\frac{K_{s}}{nZ_{r}(e^{\beta (1-s_{fc})}-1)}$$ (11)

$$\gamma =\frac{nZ_{r}}{\alpha }$$ (12)

$$\lambda^{\prime }=\lambda e^{-\Delta /\alpha }$$ (13)

Where, *S*_*h*_、 *S*_*w*_、 S* and *S*_*fc*_ are the four critical values of soil moisture, which are soil moisture hygroscopicity coefficient, wilting coefficient, vegetation water stress onset point, and field water holding capacity, respectively; *γ* is the standardized daily average precipitation(mm/d); *λ*′ is the average precipitation output frequency *d*^−1^; and the parameter *c* is a constant, obtained by solving $\int \begin{array}{l} 1\\ s_{h} \end{array}p(s)ds=1$.

In the study of this paper, the solution of the stochastic model parameters as well as the plotting of the probability density function curves are done through MWORKS 2024a software.

### 2.3. Data acquisition and processing

Soil moisture data for the maize growing season from 2020 to 2022 was collected by the pre-embedded soil monitor TDR-300 at the Wanjia Experimental Station in Harbin, basic soil physical parameters were determined by field sampling, and day-by-day precipitation data for the same period in the study area were obtained from meteorological data at the Harbin site of the National Meteorological Center of the China Meteorological Administration (http://data.cma.cn).

Experimental soil samples in the soil moisture monitor around the 2m range of sampling, soil sample collection depth of 0–20 cm, 20–40 cm, 40–60 cm, 60–80 cm four levels, each layer of the use of 100cm^3^ volume of the ring knife to take three soil samples. Ring knife soil at 105°C under the condition of drying to constant weight to calculate the soil bulk weight, another take the original loose soil, air-drying through the 2 mm sieve, through the laser particle sizer to analyze the mechanical composition of the soil, soil particle size grading standards using the international system of soil texture grading standards. Soil porosity was calculated based on the bulk weight and the density of soil particles. The maximum daily evapotranspiration of corn, Emax, can be calculated by the Penman-Monteith equation [25], *E*_*w*_, was taken as 5% of *E*_*max*_ [26]. The vegetation canopy interception threshold, *Δ*, was obtained by taking the maximum canopy interception value according to the Laio modeling assumption and referred to [27]. The rhizosphere depth, *Z*_*r*_, was set as the distribution from the surface to more than 95% of the root biomass, obtained concerning the literature by. Pan et al. [17]. The four moisture thresholds of the soil, moisture hygroscopic coefficient *S*_*h*_, wilting coefficient *S*_*w*_, water stress point S^*^, and field water holding capacity *S*_*fc*_, were correlated with the soil matrix potential, which could be obtained from the soil moisture characteristic curves, corresponding to -10 MPa, -3 MPa, -0.03 MPa, and -0.01 Mpa, respectively [14,24], The soil moisture special curve can be determined by the high-speed centrifuge method, which determines the volumetric soil water content of the soil samples then under the suction of 1 kPa, 3 kPa, 5 kPa, 10 kPa, 30 kPa, 50 kPa, 100 kPa, 300 kPa, 400 kPa, 500 kPa, 700 kPa, 900 kPa, 1000 kPa and 1200 kPa. [28] Soil saturated hydraulic conductivity *K*_*s*_ soil pore size distribution parameter *β* reference [14]. The average soil volumetric water content in the rhizosphere was calculated using the area-weighted average method [22]. The basic physical properties of the soil and the values of all the parameters involved in the Laio model are shown in **Tables 1** and **2**, respectively.

**Table 1:** Basic physical properties of soil.

Soil depth/cm	Mechanical composition/%			Texture	Dry density (g·cm^-3^)
	Clay (<0.002mm)	Silt (0.002–0.02mm)	Sand (>0.02mm)		
0–20	5.58	43.86	50.56	Sandy loam	1.29
20–40	6.46	47.91	45.63	Sandy loam	1.43
40–60	8.04	48.38	43.58	Loam	1.42
60–80	7.22	46.82	45.96	Sandy loam	1.44

**Table 2:** Model parameters and values.

symbol	Definition	unit	value
*n*	Soil porosity	-	0.47
*Z*_*r*_	Root zone depth	cm	80
*β*	Deep percolation parameter	-	13.8
*S*_*h*_	hygroscopic point	-	0.21
*S*_*w*_	Wilting point	-	0.29
S*	Water stress	-	0.71
*S*_*fc*_	Field capacity	-	0.76
*K*_*S*_	Saturated hydraulic conductivity	cm∙d^-1^	80
*Δ*	Interception capacity of vegetation	cm	0.15
*E*_*max*_	Maximum daily average evapotranspiration	cm∙d^-1^	0.372
*E*_*w*_	Soil evaporation	cm∙d^-1^	0.186
*α*	Average daily precipitation during the growing season	cm	0.371
*λ*	Average daily precipitation frequency during the growing season	d^-1^	0.485

### 2.4 Model validation metrics

In this study, the Nash-Sutcliffe coefficient of efficiency (NSE), mean absolute error (MAE), and root of mean square error (RMSE) were used for the dynamic stochastic modeling of soil moisture accuracy of the stochastic model of soil moisture dynamics was evaluated [29,30]. The formula for each index is as follows:

$$NSE=1-\frac{\sum_{i=1}^{N}{\left(p{\left(s\right)}_{model,i}-p{\left(s\right)}_{obs,i}\right)}^{2}}{\sum_{i=1}^{N}{\left(p{\left(s\right)}_{obs,i}-\bar{p{\left(s\right)}_{obs,i}}\right)}^{2}}$$ (14)

$$MAE=\frac{\sum_{i=1}^{N}\left|p{\left(s\right)}_{model,i}-p{\left(s\right)}_{obs,i}\right|}{N}$$ (15)

$$RMSE=\sqrt{\frac{\sum_{i=1}^{N}{\left(p{\left(s\right)}_{\mathrm{mod}el,i}-p{\left(s\right)}_{obs,i}\right)}^{2}}{N}}$$ (16)

Where, *p*(*s*)_*model*,*i*_ is the modeled value of the probability density function of soil moisture; *p*(*s*)_*obs*,*i*_ is the measured value; $\bar{p{\left(s\right)}_{obs,i}}$ is the mean value of the measured value; *N* is the total number of samples. NSE reflects the degree of matching between the simulated value and the measured value, the closer the value of NSE is to 1, indicating that the simulated value matches the measured value to a greater extent; MAE reflects the average absolute deviation between the simulated value and the measured value; RMSE reflects the overall error between the simulated value and the measured value.

## 3. Results

### 3.1 Precipitation patterns in the study area

**Fig 2:**
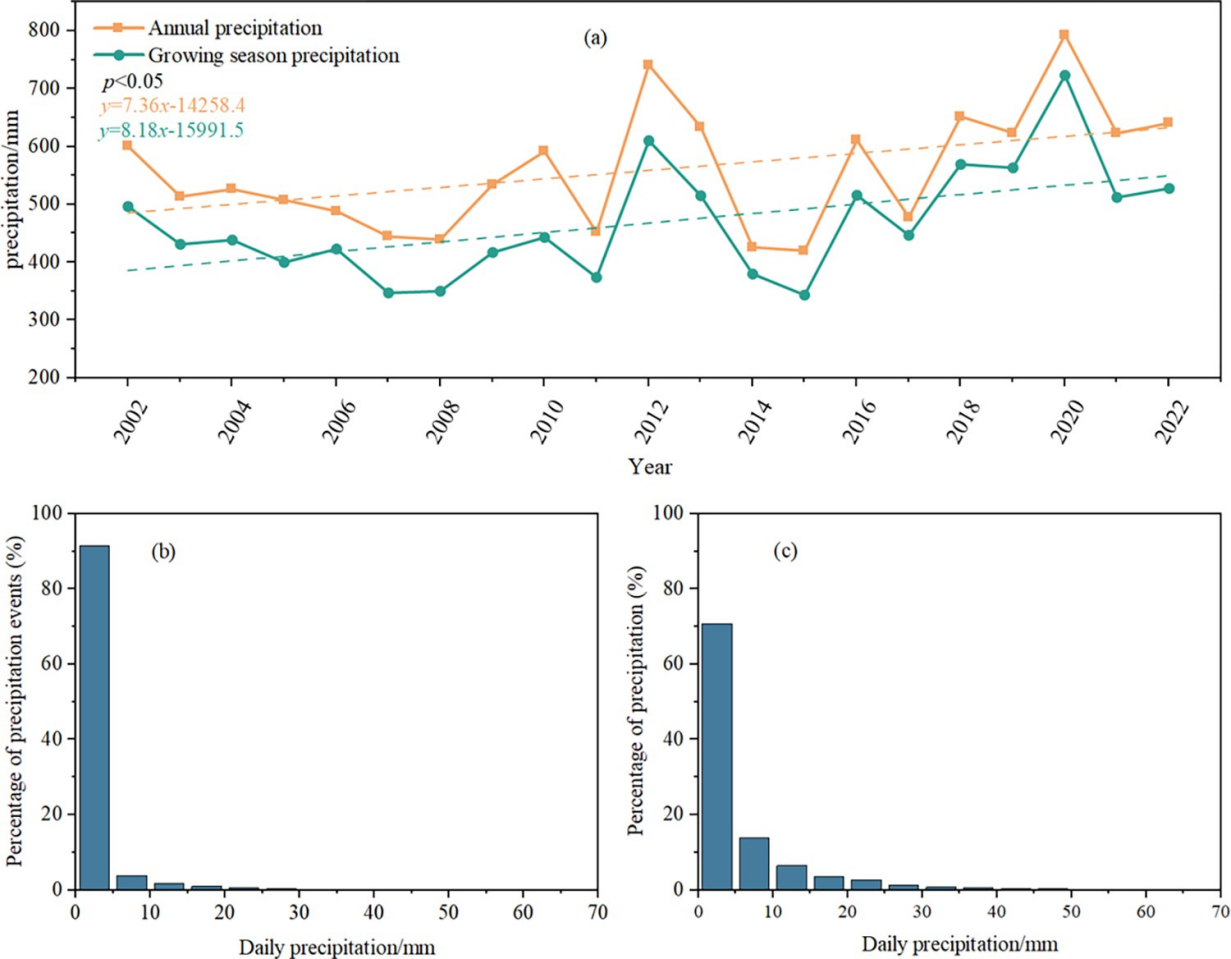
2002–2022 Precipitation patterns in the study area: (a) annual precipitation versus growing season precipitation; (b) frequency distribution of precipitation events; (c) Precipitation distribution for precipitation events.

In **Fig 2A**, it can be seen that: annual precipitation and growing season precipitation in the study area both passed the significance test (p<0.05) and showed a significant upward trend, and the linear upward trend reached 7.36 mm/a (annual precipitation series) and 8.18 mm/a (growing season precipitation series), respectively; the seasonal distribution of annual precipitation in the study area was extremely uneven, mainly concentrated in the maize growing season from May to September, accounting for an average of annual The seasonal distribution of annual precipitation in the study area was very uneven, mainly concentrated in the maize growing season from May to September, which accounted for 83.71% of the annual precipitation on average, and the highest precipitation was in August, which accounted for 19.3% of the annual precipitation on average. From **Fig 2B and 2C**, we can see the distribution characteristics of precipitation, the frequency of 0–5 mm precipitation events as well as the total amount of precipitation were the highest; the precipitation events of 0–5 mm, 5–10 mm, 10–15 mm, and >15 mm accounted for 91.57%, 3.87%, 1.71%, and 2.85%, respectively, of the annual precipitation events, and the total amount of precipitation from this precipitation event accounted for 70.77%, 13.89%, and 13.89%, respectively, of the annual precipitation. 70.77%, 13.89%, 6.49%, and 8.85% respectively, and the proportion of precipitation amount and the number of precipitation occurrences show a decreasing trend with the increase of precipitation level.

### 3.2 Characterization of soil moisture dynamics and probability distribution

#### 3.2.1 Soil moisture dynamic distribution patterns

**Fig 3:**
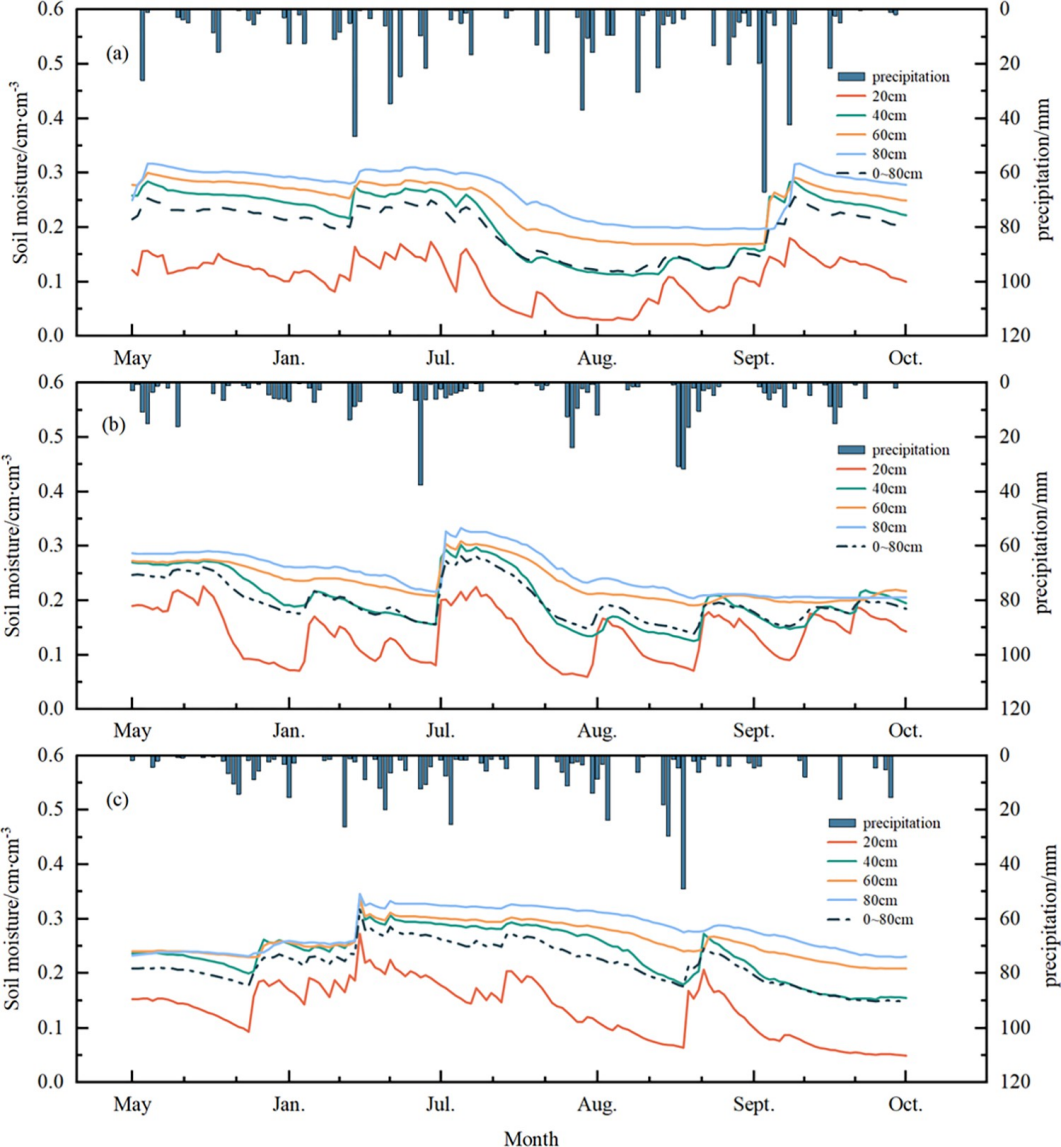
Soil water content at different depths in the root layer and daily precipitation during the growing season of maize from 2020 to 2022 in the study area:(a)2020;(b)2021;(c)2022.

Changes in soil water content at different depths during the maize reproductive period from 2020 to 2022 in the study area are shown in **Fig 3.** The trend of soil water content at different depths during the maize growing season is consistent with precipitation, and when a larger precipitation time occurs, there is a significant magnitude of increase in soil water content. The trend of soil water content at 20 cm, 40 cm, 60 cm, and 80 cm depth during the growing season and the average water content of the rhizosphere were generally consistent, and the changes in soil water content and precipitation showed consistent fluctuations due to the influence of precipitation. The magnitude of soil moisture fluctuation gradually decreased with increasing soil depth (**Fig 3**).

From the point of view of the different growth stages of corn, from May to early June for the early stage of corn growth, corn growth is slow, and precipitation increases during the same period, the soil water content remains at a high level. Early June to late July into the rapid development of maize, soil water content showed a trend of slow increase. late July to mid-August growth in the middle, the corn entered the critical stage of growth and development, water consumption increased, soil water content slowly decreased. mid-August to the end of September corn growth in the late stage, and water consumption decreases, but precipitation during the same period decreases as well, soil water content gradually stabilized.

The statistical characteristics of soil moisture in the study area are shown in **Table 3**, the average water content of the whole soil layer in the growing season averaged over the years was 0.203, the coefficient of variation was 0.129, and in terms of inter-annual variations, the average water content of the whole soil layer in 2022 was the highest, which was 0.206, and the coefficient of variation was the lowest, which was 0.184. The variation of the soil moisture content at different depths ranged from 0.069 to 0.319, and the minimum and maximum values appeared at 20cm and 80cm, respectively. The minimum and maximum values occurred at 20cm and 80cm, respectively, and the mean values varied from 0.126 to 0.265, with coefficients of variation ranging from 0.103 to 0.218, all of which were moderately variable (0.1 < CV < 1). From 20 cm to 80 cm, the mean value of soil water content gradually increased, but the coefficient of variation showed a decreasing trend, indicating that with the increase of soil depth, the fluctuation amplitude of soil water content was weakened, and the state became more and more stable, and less affected by precipitation and evapotranspiration. In addition, the skewness of soil moisture in each layer fluctuated between -0.056 and 0.239 and the kurtosis fluctuated between 1.895 and 2.236 during the growing season. The P-value of soil moisture in each layer by the K-S test was greater than 0.05, obeying a normal distribution [31].

**Table 3:** Characteristics of soil water content at different depths during the maize growing season from 2020 to 2022 in the study area.

Year	Soil depth/cm	Soil volumetric water content /cm^3^∙cm^-3^				SD	CV	K-S	Skewness	Kurtosis
		Maximum	Minimum	Average	Median					
2020	20	0.180	0.03	0.105	0.117	0.041	0.387	0.118	-0.467	2.077
	40	0.284	0.111	0.211	0.242	0.059	0.279	0.214	-0.583	1.634
	60	0.300	0.167	0.241	0.264	0.047	0.196	0.223	-0.609	1.624
	80	0.317	0.196	0.266	0.287	0.043	0.164	0.234	-0.607	1.662
	0–80	0.254	0.109	0.189	0.207	0.046	0.246	0.187	-0.543	1.680
2021	20	0.226	0.06	0.138	0.143	0.046	0.334	0.116	0.063	1.782
	40	0.302	0.126	0.203	0.191	0.049	0.240	0.122	0.410	1.984
	60	0.309	0.191	0.234	0.224	0.034	0.143	0.152	0.576	2.041
	80	0.333	0.204	0.249	0.242	0.039	0.154	0.134	0.446	1.984
	0–80	0.276	0.128	0.187	0.180	0.036	0.190	0.156	0.651	2.377
2022	20	0.273	0.049	0.135	0.145	0.051	0.379	0.094	-0.085	2.024
	40	0.341	0.152	0.237	0.241	0.046	0.195	0.091	-0.315	2.052
	60	0.336	0.209	0.260	0.253	0.031	0.118	0.123	0.177	1.897
	80	0.346	0.230	0.279	0.277	0.036	0.129	0.145	0.119	1.449
	0–80	0.310	0.138	0.206	0.207	0.038	0.184	0.056	-0.052	2.279
2020–2022 Multi-year average	20	0.176	0.069	0.126	0.125	0.027	0.218	0.070	-0.197	2.169
	40	0.281	0.151	0.217	0.223	0.035	0.161	0.105	-0.140	1.971
	60	0.294	0.200	0.245	0.251	0.025	0.103	0.103	-0.056	1.895
	80	0.319	0.224	0.265	0.268	0.025	0.094	0.077	0.239	2.236
	0–80	0.256	0.154	0.203	0.206	0.026	0.129	0.112	0.033	1.900

#### 3.2.2. Characterization of relative soil moisture probability distribution

**Fig 4:**
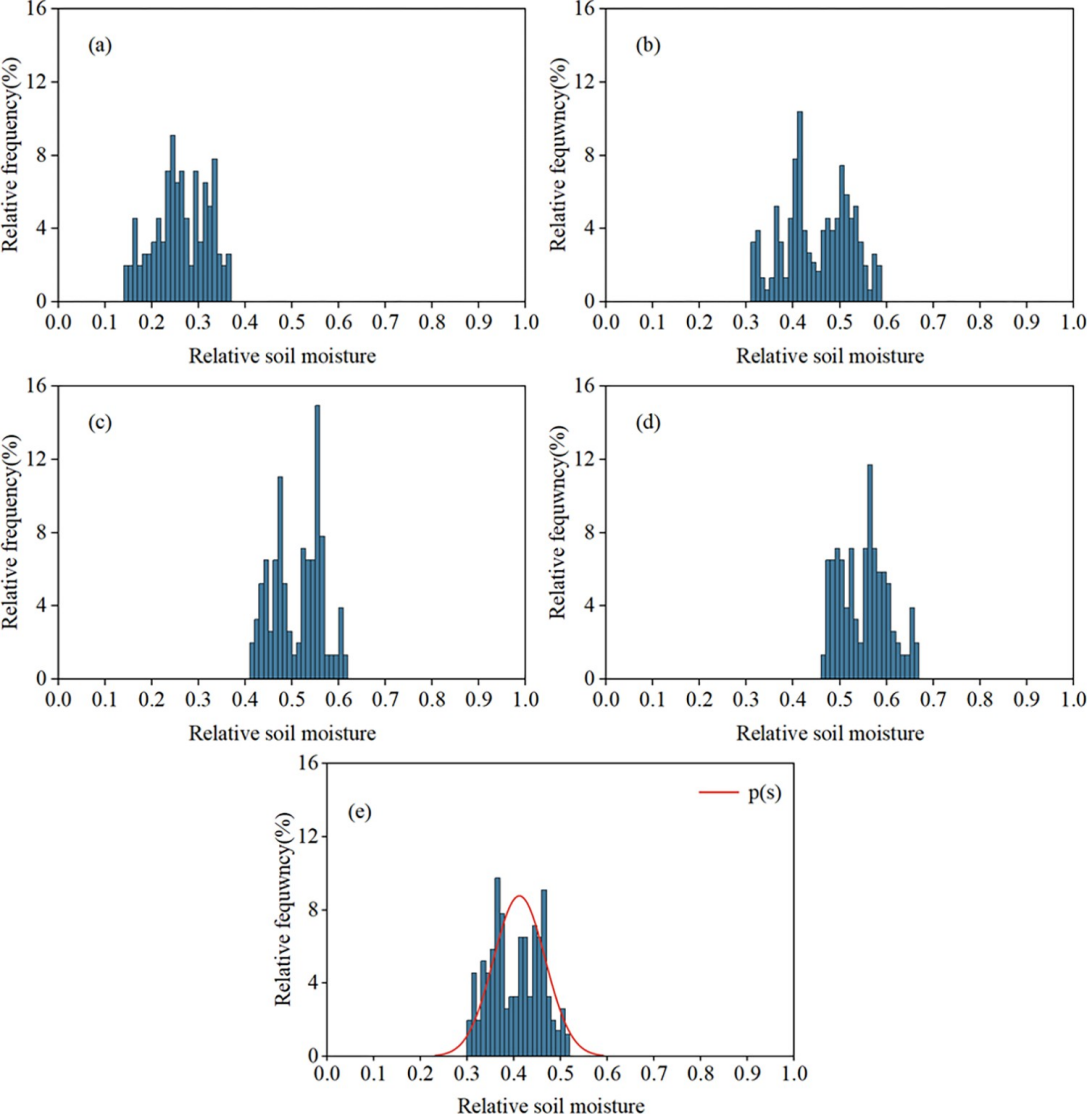
Probability distribution of soil moisture at different depths during the growing season 2020–2022 in the study area: (a)20cm;(b)40cm;(c)60cm;(d)80cm;(e)0–80cm root layer Zr.

The probability distribution of relative soil water content at different depths and rhizosphere during the 2020–2022 multi-year growing season is shown in **Fig 4**. The histogram of the probability distribution of relative soil water content at 20 cm was in the shape of a single peak, with the peak occurring at *s* = 0.25 and the width of the distribution ranged from 0.14 to 0.37; among them, the relative soil water content was mainly concentrated in the range of 0.2 to 0.34, which accounted for 77.27% of the total amount; the distribution probability was highest in the range of 0.24–0.25, which accounted for 9.09% of the total amount, and the distribution probability was lowest in the range of 0.14 to 0.15 is the lowest, accounting for 1.94% (**Fig 4A**). The peak of the relative soil moisture probability distribution at 40 cm occurred at s = 0.42, and the breadth of the peak was the widest among all the soil depths, ranging from 0.31 to 0.59; the highest distribution probability of 0.41 to 0.42 was 10.38%, and the lowest of 0.56–00.57 with a percentage of 0.65% (**Fig 4B**). The probability distribution of soil moisture at 60 cm with compared to 20 cm and 40 cm, the position of the peak is shifted to the right, occurring at *s* = 0.56, and the width of the distribution was also narrower, at 0.21. **(Fig 4C**); it can be seen that the probability distribution of soil moisture at 80 cm is more centralized compared to other depths, and the width of the distribution is being in the range of 0.46–0.67; of which 0.41–0.42 distribution probabilities are highest, and 0.56–0.57 lowest, with a proportion of 0.64%. (**Fig 4D**). From 20cm to 80cm, with the increase in depth, the probability distribution of soil moisture gradually moved to the right side of the wet place, and the position of the peak also gradually moved to the right. It can be seen that the average soil moisture probability distribution in the rhizosphere from 0 to 80 cm was in the form of a single peak, which was more concentrated compared to that at different depths; its peak occurred at *s* = 0.37, and the distribution width ranged from 0.30 to 0.52, but it was mainly concentrated in the range of 0.37 to 0.47, with a probability distribution of 58.84%; among them, the highest probability distribution was at 0.36 to 0.37 with 10.03%, and 0.51–0.52 is the lowest with 1.19% (**Fig 4E**).

### 3.3 Stochastic modeling of soil moisture dynamics

**Fig 5:**
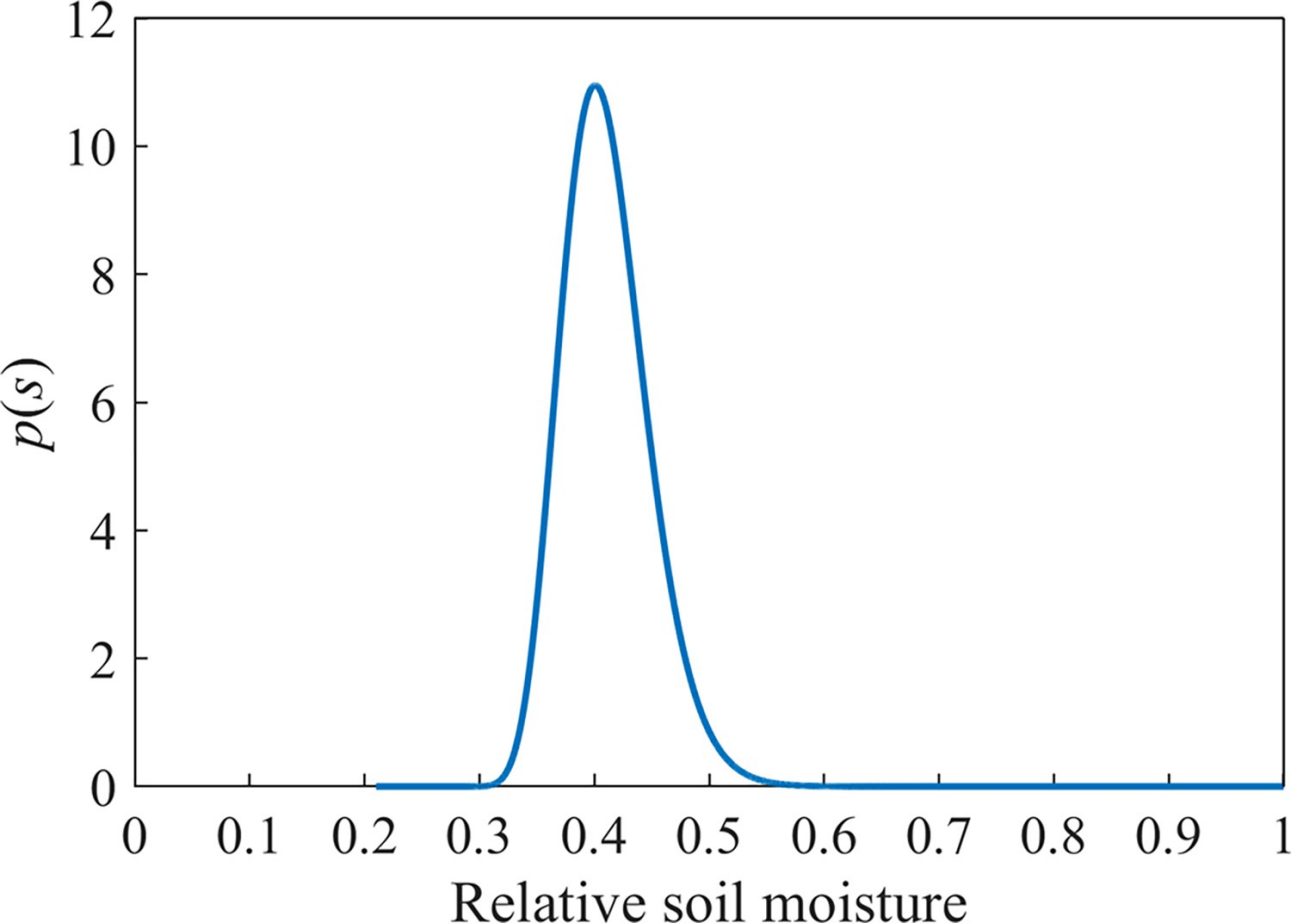
Probability density function of soil moisture obtained from simulation of spring corn growing season in the study area from 2020 to 2022.

The precipitation parameters from 2020 to 2022 in Table 2 and other parameters involved in the model were brought into Eq (8) as input terms of the Laio stochastic model to obtain the soil moisture probability density function plots at one point in the rhizosphere during the growing season of maize in the study area (**Fig 5**). As can be seen from **Fig 5**, the corn *p(s)* simulated by the stochastic model of soil moisture was single-peaked with a peak of 10.95, the peak occurred at *s* = 0.40, and the broadness of the peak was distributed in the range of 90% confidence interval [0.35,0.47]. The measured *p(s)* peak for corn was 8.77, corresponding to a relative soil water content of *s* = 0.411, distributed in the range of 90% confidence interval [0.30,0.53]. The location where the simulated *p(s)* peak appeared was the same as the measured one, with a difference of 0.011; compared with the location where the simulated *p(s)* peak appeared, the simulated *p(s)* peak differed from the measured one by 2.18, with a slight decrease in accuracy. The distribution intervals of the simulated *p(s)* relative soil moisture were very different from the measured values, with the minimum and maximum values differing by 0.05 and 0.06, respectively. The difference between the simulated and measured values is analyzed, and the results are shown in **Table 4**. The NSE, MAE, and RMSE values of the model were 0.86, 0.58, and 0.95, respectively, indicating that the Laio stochastic model was able to make good predictions of the stochastic distribution characteristics of soil moisture in the rhizosphere (0–80 cm) of the maize growing season in the study area.

**Table 4:** Analysis of simulated and measured soil moisture probability density function for maize growing season in the study area.

Vegetation	Value	Characteristic value of soil moisture				NSE	MAE	RMSE
		Maximum	Minimum	Peak	Broadness			
Corn	Simulated	0.47	0.35	10.95	0.12	0.86	0.558	0.95
	Measured	0.53	0.30	8.77	0.23			

### 3.4. Parameter sensitivity analysis of a stochastic model of soil moisture dynamics

**Fig 6:**
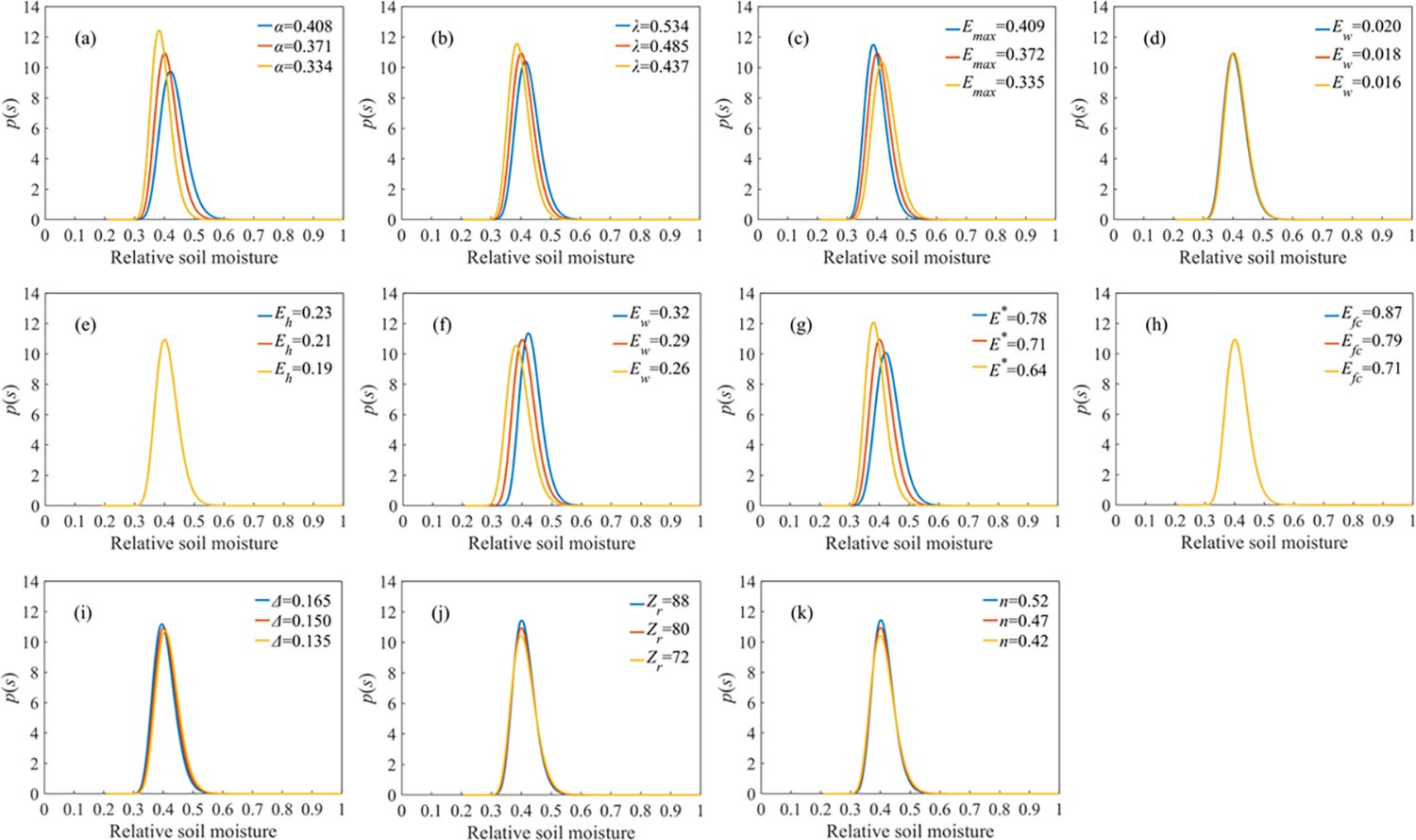
Sensitivity analysis plot of model parameters.

Sensitivity analysis can generally be divided into local sensitivity analysis and global sensitivity analysis [32], this study uses the local sensitivity analysis method to carry out sensitivity analysis on the parameters involved in the Laio model, change the value of a parameter in the model (increase or decrease by 10%), and the rest of the parameters are kept unchanged, and get the probability density curve of soil moisture, *p(s)* as shown in **Fig 6**. Comparing the curves in the **Fig 6**, it can be found that when the parameters *α*, *λ*, *E*_*max*_, *S*_*w*_, S^*^ are increased or decreased by 10%, the peak value of the *p(s)* curve as well as the position of the peak change more obviously, with the change range of 10% to 15%, while the broadness of the peak basically remains unchanged; among them, when *α*, *λ*, S^*^ and *E*_*max*_ gradually increase, the position of the peak moves to the left and the peak value increases gradually; when *S*_*w*_ gradually increases, The position of the peak moves to the right and the peak gradually increases; when the parameters *Δ*, *Z*_*r*_ and *n* increased or decreased by 10%, the change of the peak of the *p(s)* curve and the position of the peak is very small, below 5%, and the broadness of the peak does not change; when the parameters *E*_*w*_, *S*_*h*_, *S*_*fc*,_ etc., are increased or decreased by 10%, the peak of the *p(s)* curve, the position of the peak and the broadness of the peak remain unchanged. From the degree of influence of different parameters on the shape of the soil moisture probability density function curve *P(s)*, it can be found that the model parameters *α*, *λ*, *E*_*max*_, *S*_*w*_, and S^*^ have the greatest influence on the soil moisture probability density curve, and the sensitivities of these parameters are *α*, S^*^, *S*_*w*_, *E*_*max*_, and *λ* in descending order, and they mainly affect the peaks and the locations of the peaks of the *P(s)* curve.

## 4. Discussion

### 4.1. Precipitation patterns and their impact on soil water content

Precipitation has a significant effect on the changing characteristics of soil moisture, which is one of the important sources of soil moisture replenishment [33]. Guo et al. [34] studied the relationship between soil moisture and precipitation in the Yellow River Basin and found that soil moisture was positively correlated with precipitation, and there was a certain lag time between the effects of precipitation and temperature on soil moisture, with a lag time of about 1–2 months. Similarly, in this study can also be found in soil moisture and precipitation in a certain degree of positive correlation, with the increase of precipitation, soil moisture also tends to increase, and soil moisture changes lagged behind precipitation for a certain period of time, but after that the time is about 1–2 days, which may be caused by different time scales in the study to the time in this paper soil humidity is the time is the day scale. Mimeau et al. [35] studied the effect of precipitation characteristics (precipitation interval, intensity) on soil moisture changes in the Mediterranean region of France and found that soil moisture is more sensitive to intermittent precipitation and less sensitive to precipitation intensity. However, in this paper it is clear that precipitation intensity has a greater influence on soil moisture changes, with each sudden change in soil moisture following a high-intensity precipitation event, which may be due to climatic differences between the study areas. The effect of small precipitation events on the shallow soil water volume (0–20 cm) was more obvious, and the large precipitation events significantly affected the water content of the entire soil layer, so the shallow soil water volume fluctuated more significantly under small precipitation events compared to the deeper soil, which was in line with the results of the study by Dong et al. [36].

### 4.2 Probability density function of soil moisture in the rhizosphere during the growing season

Parameter sensitivity analysis can provide a basis for the parameter rate setting and model application of the model in different situations, which is an important link in model simulation [37]. Laio et al.[14] analyzed the shape of *p(s)* under different climatic factors, precipitation characteristics, soil types, rhizosphere depths, and evapotranspiration, and found that, with the increase of rhizosphere depth *Z*_*r*_ increased, the peak of *p(s)* shifted to the right, and the size of the peak did not change significantly; from wet to arid climates, the peak of *p(s)* increased significantly; the peak of *p(s)* and the width of the distribution of *p(s)* in wet climates were mainly concentrated at higher soil water content, and the width of the distribution of p(s) was wider than that in arid conditions. As the intensity of climatic fluctuations increases from year to year, the distribution width of *p(s)* increases, and the phenomenon of bimodal peaks occurs, with the newly emerging bimodal peaks concentrated in both the dry and wet directions [38,39]. However, the *p(s)* in this study were unimodal and did not show a bimodal phenomenon, mainly because the intensity of interannual climatic fluctuations during the growing season in the study area was not drastic. The peak value of soil moisture *p(s)* in the growing season of maize obtained from the simulation in this study was 10.95, which occurred at *s* = 0.40, and the width of the distribution ranged from 0.34 to 0.49, whose shape was close to the shape of the soil moisture *p(s)* curve obtained by Suo et al. [40] using the Laio stochastic model with the rest of the parameters remaining unchanged and only changing the model parameter S^*^ to 0.51. The *p(s)* curves obtained by Pan et al.[17] using the Laio and Rodriguze-Iturbe models, respectively, to stochastically simulate the soil moisture dynamics of the maize rhizosphere in the North China Plain region was much larger in the location of the peaks’ appearance and the breadth of the peaks than the results of the present study, whereas the *p(s)* curve obtained by Yin et al. [41] for the analysis of the rhizosphere in the southern dunes of the Gulbantong Desert during the growing season was much larger than the results of the present study. The *p(s)* curves, the location of the peak, and the broadness of the peak are again much smaller than the results of this paper, mainly because the precipitation intensity in this study area is lower than that in the North China Plain area and the irrigation factor is not taken into account, but the precipitation intensity is higher than that in the desert area. With global warming and increased precipitation, the soil moisture probability density function curve *p(s)* in this study area may move in the wet direction, and even a double-peak phenomenon may occur, but the increase in temperature will also increase *E*_*max*_ in the output term of the model, so that the probability density curve *p(s)* will move to the left in the direction of dryness, offsetting some of the effects brought by the increase in precipitation, but how the *p(s)* curve will in practice However, how the *p(s)* curve will change in practice is still to be further studied, which is of great significance for the rational arrangement of crop planting in agricultural production.

### 4.3. Sensitivity analysis of model parameters

Due to the insufficient understanding of the soil moisture cycle mechanism and the excessive number of parameters involved in the model, resulting in large uncertainties in the model, which affects the simulation accuracy of the model [42], it is necessary to analyze the sensitivity of the model parameters. Yao et al. [24] analyzed the parameter sensitivity of the Laio model for the growing season in the horqin sandland and classified the model’s sensitivity to the parameters into three levels: weakly sensitive parameters, moderately sensitive parameters, and strongly sensitive parameters, in which *α*、*λ*、*E*_*max*_、*S*_*w*_ and S^*^ had the greatest impact on the *p(s)* curve, and *K*_*s*_ had the least impact. Li et al. [43] analyzed the parameter sensitivity of a stochastic model of soil moisture dynamics in the sandy gravel plains and dunes of the Namibia Desert, Afghanistan. Parameter sensitivity analysis of a stochastic model of soil moisture dynamics, divided all parameters into bounded (*n*、*S*_*h*_、*S*_*w*_、S^*^ and *S*_*fc*_) and unbounded (*Z*_*r*_、*E*_*max*_、*E*_*w*_ and *K*_*s*_) groups, quantified the sensitivity of soil and vegetation parameters to the stochastic model, and found that the model was more sensitive to the parameters of the bounded group, with average sensitivities ranging from 0.00011% to 44%, whereas the average sensitivities for the parameters of the unbounded group were average sensitivity was only -0.00065%–0.07% and the sensitivity of the parameters was negative except for the parameter *n* of the unbounded group in the gravel plain. Compared with the study of Yao et al. [24]. The maximum sensitivity parameters of the Laio stochastic model in this study are all *α*、*λ*、*E*_*max*_、*S*_*w*_ and S^*^, and the change in the peak value of *p(s)* is only 10–15% when increasing or decreasing the values of these parameters by 10%, but the change in the peak value of *p(s)* is more than 60% when increasing or decreasing the values of these parameters in Yao et al. [24]. In the study of Li et al. [43], the parameter with the strongest model parameter sensitivity is *S*_*h*_, but in this paper, the parameter *S*_*h*_ is one of the least sensitive parameters and has no effect on the model output results. These differences may be caused by the fact that the study area of this paper is mainly in plain farmland, while the previous researchers’ analysis of model sensitivity is mainly in desert dunes and other places, and the vegetation, soil, and other factors are different in different study areas.

## 5. Conclusions

Precipitation in the study area has shown a significant increasing trend in the last two decades and in terms of temporal distribution pattern, the precipitation is mainly concentrated in the growing season, which provides abundant rainfall resources for maize growth in the area. Soil water content showed obvious variability at different periods of the growing season of maize in the study area, maintained at a high level from May to early June, declined rapidly from early June to July, and stabilized from August to September, it showed some random characteristics in the inter-annual variation. Soil surface water content (0–20cm) was greatly affected by precipitation and evaporation, and soil moisture fluctuated significantly; with the increase in depth, the degree of fluctuation weakened, and the coefficient of variation decreased from 0.218 (20cm) to 0.094 (80cm).

Parameters *α*、*λ*、*E*_*max*_、*S*_*w*_ and S^*^ have the highest sensitivity, mainly affecting the peaks of the *p(s)* curves and the positions of the peaks, and the peaks of the *p(s)* gradually increase when the values of these parameters increase; parameters *Δ、Z*_*r*_ and *n* have lower sensitivities, which have less influence on the shape of the *p(s)*; parameters *E*_*w*_、*S*_*h*_、*S*_*fc*_ and so on have the lowest sensitivities, which do not affect the shape of the *p(s)*.

The *p(s)* peak size, peak location, and peak broadness simulated by the Laio soil moisture dynamic stochastic model were very close to the measured results, in which the simulation of peak size and peak location was better, and the Nash efficiency coefficients (NSE), mean absolute error (MAE), and root mean square error (RMSE) were 0.86, 0.58, and 0.95, respectively, which indicated that the Laio model had good applicability in the the applicability of soil moisture probability density function in the rhizosphere of maize growing season in the black soil area of Songnen Plain is better, and it can be used to simulate the dynamic stochastic characteristics of soil moisture in farmland in this area, predict soil moisture, and provide a theoretical basis for the efficient utilization of soil moisture in farmland in this area.
